# Variability in the estimation of ungulate group sizes complicates ecological inference

**DOI:** 10.1002/ece3.6463

**Published:** 2020-06-10

**Authors:** Herbert Kasozi, Robert A. Montgomery

**Affiliations:** ^1^ Research on the Ecology of Carnivores and their Prey (RECaP) Laboratory Department of Fisheries and Wildlife Michigan State University East Lansing MI USA.

**Keywords:** behavioral ecology, group living, group size, social behavior, ungulates

## Abstract

1. Foundational work has examined adaptive social behavior in animals in relation to the costs and benefits of group living. Within this context, a “group” of animals represents an organizational unit that is integral to the study of animal ecology and evolution.
2. Definitions of animal group sizes are often subjective with considerable variability within and across species. However, investigations of both the extent and implications of such variability in the estimation of animal group sizes are currently lacking.
3. Selecting ungulates as a case study, we conducted a literature review to assess prevailing practices used to determine group sizes among terrestrial Cetartiodactyla and Perissodactyla. Via this process, we examined group size definitions for 61 species across 171 peer‐reviewed studies published between 1962 and 2018.
4. These studies quantified group sizes via estimation of ungulate aggregations in space and time. Spatial estimates included a nearest neighbor distance ranging from 1.4 m to 1,000 m, and this variation was partially explained by a weak positive correlation (|*r*| = .4, *p* < .003) with the body size of the ungulate research subjects. The temporal extent over which group size was estimated was even broader, ranging from three minutes to 24 hr.
5. The considerable variability in ungulate group size estimation that we observed complicates efforts to not only compare and replicate studies but also to evaluate underlying theories of group living. We recommend that researchers: (a) clearly describe the spatiotemporal extents over which they define ungulate group sizes, (b) highlight foundational empirical and ecological rationale for these extents, and (c) seek to align such extents among individual species so as to facilitate cross‐system comparisons of ungulate group size dynamics. We believe an integrative approach to ungulate group size estimation would readily facilitate replication, comparability, and evaluation of competing hypotheses examining the tradeoffs of animal sociality.

## Graphical abstract

We conducted a literature review to assess prevailing practices used to determine group sizes among ungulates. These practices were highly variable including estimation of aggregations in space and time. This variability and lack of coherence complicates efforts to not only compare and replicate studies but also evaluate underlying theory of group living.

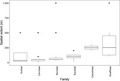

## 1 INTRODUCTION

Coarsely, animal sociality refers to the tendency of animals to live in groups (Dunbar & Shultz, 2010; Krause, Lusseau, & James, 2009; Seebacher & Krause, 2017; Sih, Hanser, & McHugh, 2009). Research on animal social behavior lies at the cornerstone of the fields of evolutionary biology and behavioral ecology (Alexander, 1974; Cleve & Akçay, 2014; Grodwohl, 2017; Hamilton, 1964; Sterck, Watts, & van Schaik, 1997). Social animals form and live in groups mostly comprised of conspecifics (Krause & Ruxton, 2002; Reiczigel, Lang, Rozsa, & Tothmeresz, 2008) and to a lesser extent phylogenetically close heterospecifics (Periquet et al., 2010; Schmitt, Stears, & Shrader, 2016; Schmitt, Stears, Wilmers, & Shrader, 2014). Thus, a *group* represents some unit of social animals whereas *group size* is a metric used to quantify that unit (Ofstad, Herfindal, Solberg, & Sæther, 2016; Reiczigel et al., 2008; Salazar, Waldner, & Stookey, 2016).

Animal group dynamics are critical to ecological and evolutionary processes including mating, foraging, migration, dispersal, and predator protection, among others (Estes, 1974; Geist, 1974; Jarman, 1974; Ruckstuhl & Neuhaus, 2000, 2002, 2009; Seebacher & Krause, 2017; Sterck et al., 1997; White, Cameron, & Peacock, 2010). Some costs of group living include higher levels of predator attack due to increased predator detection and encounter rates (Alexander, 1974; Focardi & Paveri‐Fontana, 1992; Krause & Ruxton, 2002; Sterck et al., 1997), increased competition for resources (Clutton‐Brock & Coulson, 2002; Janson, 1988; Jarman, 1974; Ruckstuhl & Neuhaus, 2009), and higher levels of parasitic and disease transmission (Wal, Paquet, & Andres, 2012). These tradeoffs of group living influence fission‐fusion dynamics with important implications for animal group sizes across spatial and temporal dimensions (Aurelli et al., 2008; Dunbar & Shultz, 2010; Krause & Ruxton, 2002). Thus, group sizes reflect the adaptation of animals to environmental and social conditions (Geist, 1974; Seebacher & Krause, 2017; Smith, 1964).

Group living has been extensively studied in birds (Nagy, Zsuzsa, Dora, & Tamas, 2010), mammals (Ahmad et al., 2018; Body, Weladji, Holand, & Nieminen, 2015a, 2015b), fish (Katz, Tunstrøm, Ioannou, Huepe, & Couzin, 2011; Oboshi, Shohei, Atsuko, & Hidenori, 2003), and insects (Buhl et al., 2006; Foster & Treherne, 1981). Among mammals, several studies have analyzed the short‐term dynamics of group fusion‐fission across species (e.g., Barrette, 1991; Caughley, 1964; Gerard, Bideau, Maublanc, Loisel, & Marchal, 2002; Gueron & Levin, 1995; Marchal, Gerard, Boisaubert, & Bideau, 1998; Pays, Benhamou, Helder, & Gerard, 2007). Such dynamics have adaptive functional consequences including protection of young, resource use, intraspecific competition for resources, as well as predation and predator avoidance (Crook, Ellis, & Goss‐Custard, 1976). Thus, social cohesion is an important determinant of fitness for group‐living species (Silk, 2007). Ultimately, the cumulative effects of social interactions among group members may lead to long‐term adaptations with evolutionary implications (Dunbar & Shultz, 2010; Grodwohl, 2017). For example, dominance hierarchies among males for access to mates may lead to the evolution of sexually selected traits (Qvarnström & Forsgren, 1998). In the same light, sociality across certain prey species represents an evolutionary tradeoff between adaptations to avoid predation and those associated with maximizing the consumption of critical resources (e.g., Bowyer, McCullough, & Belovsky, 2001).

Considerable research has investigated the mechanisms that influence animal group formation (see Aurelli et al., 2008; Dunbar & Shultz, 2010; Gerard et al., 2002; Krause & Ruxton, 2002). Such activities are inherently challenging given fission‐fusion social dynamics and spatial variation in density of the animal subjects (Barrette, 1991; Monteith, Sexton, Jenks, & Bowyer, 2007; Reiczigel et al., 2008). These challenges have led to a diversity of group size estimation techniques across ecological studies (see Krause & Ruxton, 2002). However, an understanding of the magnitude and consequences of such variability in animal group size definitions is currently lacking. This is particularly apparent among ungulates, a diverse taxonomic group representing some of the world's most social mammals (Groves & Grubb, 2011; Zurano et al., 2019).

Here, we used research on terrestrial species in the taxonomic orders Cetartiodactyla and Perissodactyla as a case study and conducted a literature review of studies referencing ungulate group size estimation. We synthesized the ways in which researchers estimated ungulate group sizes, highlighted the extent of methodological variation prevalent in the protocols employed, and examined the implications of this variability for inference and comparability across research sites and species. A group is a critical concept underlying animal socio‐ecological and evolutionary research (Krause & Ruxton, 2002). Thus, our reflections on the prevailing practices in ungulate group size estimation are critical for future socio‐ecological and evolutionary research on these species with subsequent implications for other group‐living animals.

## 2 METHODS

### 2.1 Ungulates

**FIGURE 1:**
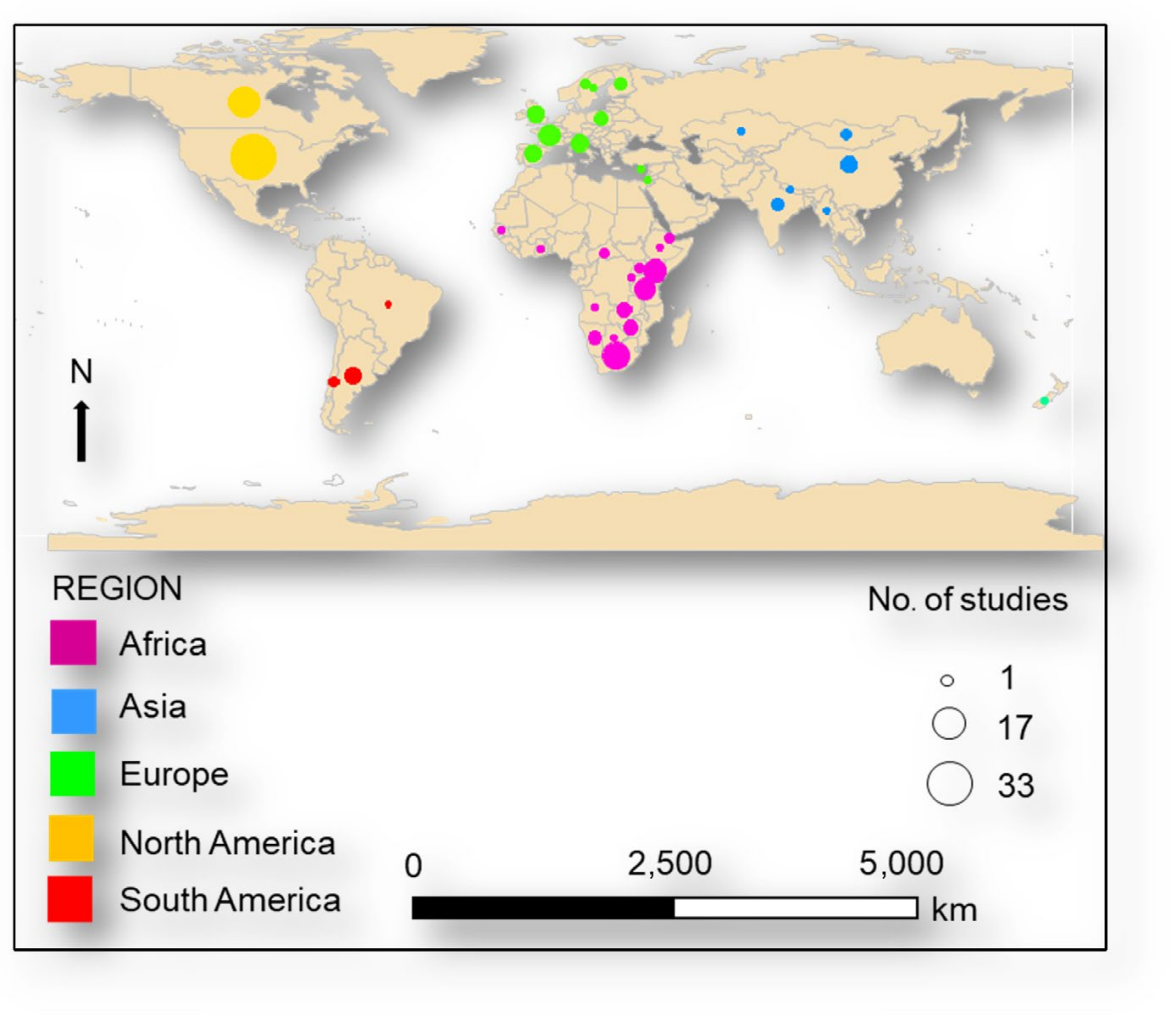
Spatial extent of the field study locations featured among 171 studies, published between 1962 and 2018, assessing ungulate group size dynamics

Given that ungulates are among the most social and diverse mammal radiations (Groves & Grubb, 2011; Zurano et al., 2019), we framed our analysis to include studies of free ranging, naturally, or seminaturally occurring terrestrial species in the orders Cetartiodactyla and Perissodactyla. Ungulates in these orders inhabit a wide range of habitats around the world from the Sudanic grassland ecosystems of Africa to the boreal forests of the northern hemisphere (Figure 1; Groves & Grubb, 2011). This spatial diversity is matched by reciprocal diversity in behavioral traits among ungulate species resulting in a range of grouping patterns (Groves & Grubb, 2011; Ofstad et al., 2016).

### 2.2 Literature review

In August of 2018, we conducted a literature review examining ungulate group size definitions. We used the Web of Science, Scopus, Wildlife Studies Worldwide, and the Michigan State University libraries search engines to conduct this review. To obtain as many peer‐reviewed studies as possible across all search engines, we used a combination of terms via a multi‐step process including primary, secondary, and tertiary searches. We used “ungulate AND group size AND group dynamics OR herd size OR herd composition AND sociality OR group living” as our primary search terms. We then combined these primary search terms with “fusion‐fission OR aggregation OR population structure OR social structure OR herbivore behavior” in the secondary search. Finally, in the tertiary search we combined the primary and secondary search terms with “predation risk OR group associations OR plasticity OR seasonal grouping OR social bonds.” We included studies that assessed group dynamics for both conspecific and heterospecific interactions. We discarded studies that did not assess ungulate grouping patterns, those that involved domesticated ungulates, and studies of recently reintroduced ungulates. We did so based on the consideration that domesticated and recently reintroduced ungulates may not exhibit natural grouping patterns.

### 2.3 A framework for ungulate group size estimation

**FIGURE 2:**
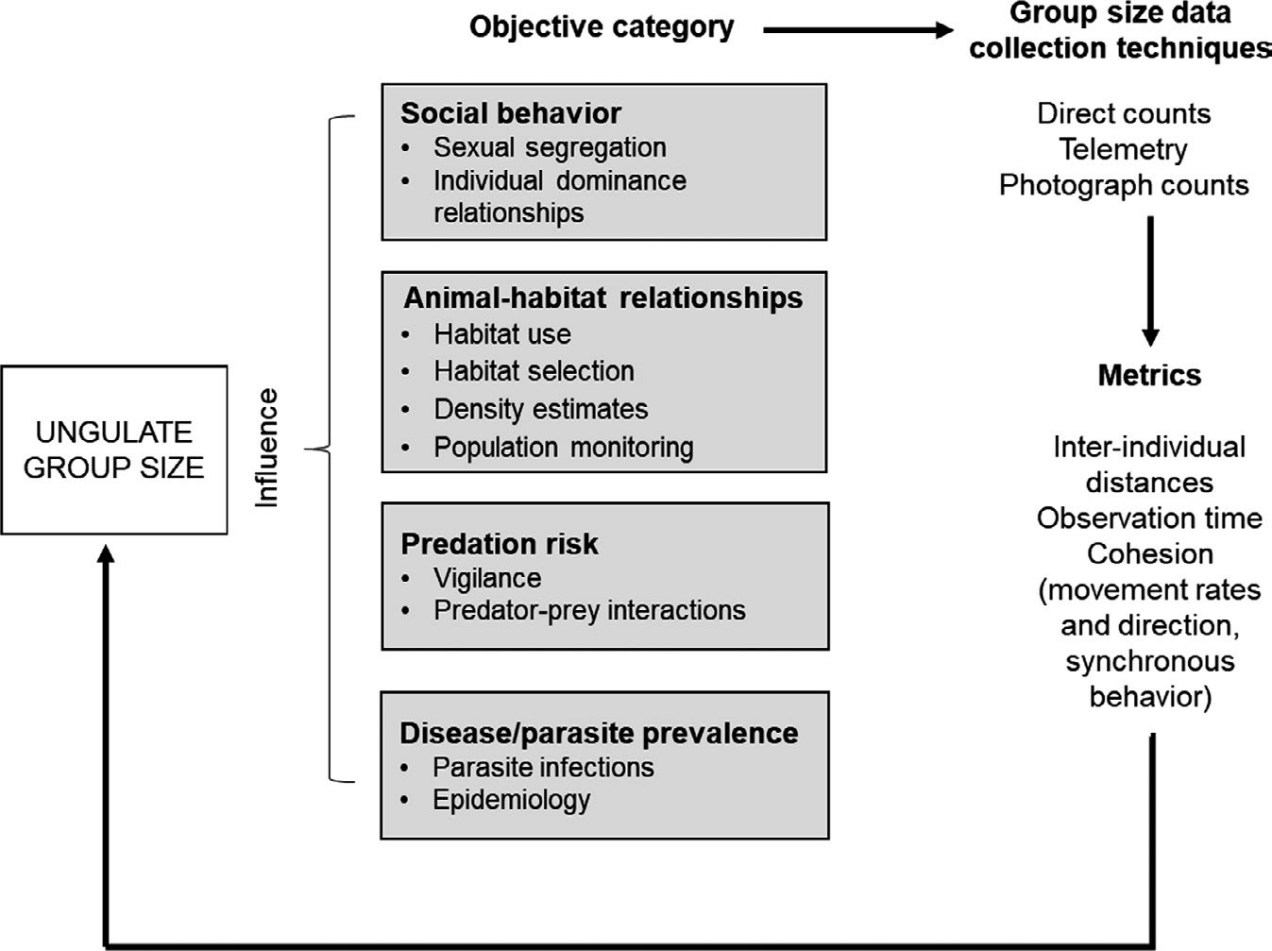
A framework for ungulate group size estimation. Studies describing ungulate group sizes fall into one of four standardized objective categories. The study objectives determine the field techniques for group size estimation. The field techniques used for group size estimation determine the metrics employed and ultimately the group sizes reported by studies. Arrows depict the sequence of events that lead to observed ungulate group sizes

Scientific studies of social ungulates often require some enumeration or description of ungulate group sizes. We compared definitions of ungulate group sizes as inferred from each study's research objectives and methodological descriptions. We placed the studies in one of four standardized objective categories to compare group size definitions (Figure 2). These objective categories included (a) social behavior, (b) animal‐habitat relationships, (c) predation risk, and (d) disease/parasite prevalence. We then recorded all metrics used to define ungulate groups across all species, data collection techniques, and study objectives (Figure 2; Table 1).

**TABLE 1:** Description of metrics used to define ungulate groups from a review of 171 studies, published between 1962 and 2018, assessing ungulate group size dynamics

Metric	Description	References
Distance/Spatial extent	Interindividual distance among group members	Bowyer et al. (2001); Carter, Seddon, Frère, Carter, and Goldizen (2013)
Time	Observation time bound used to determine group membership during field observation or telemetry studies	White et al. (2010); Koen, Tosa, Nielsen, and Schauber (2017)
Cohesion	All activities and behaviors that promote fusion among group members, for example, movement, mating, and foraging.	Barja and Rosellini (2008), Salazar et al. (2016)

### 2.4 Relationship between body size and spatial extent

Body size is an important factor that influences ecological processes across spatiotemporal scales (Smith & Lyons, 2011). Furthermore, there are three orders of magnitude between the smallest and largest terrestrial species in the taxonomic orders Cetartiodactyla and Perissodactyla. Thus, we hypothesized that the relative body size of the ungulate research subjects might correlate with the spatial extent used to define their group sizes across studies. We obtained body mass data of all species included in our study from the Phylogenetic Atlas of Mammal Macroecology (Faurby et al., 2019). This database contains the most up‐to‐date trait data for all 5,831 known mammal species from the last interglacial period (~130,000 years ago) until present (Faurby et al., 2018). We then used the nonparametric Spearman's rank correlation test to examine the correlation between body size and spatial extent of group size estimation.

## 3 RESULTS

In total, our literature review returned 534 studies. Upon review, we eliminated 363 studies from consideration given that they did not meet our criteria for inclusion in the analysis, as described in the methods. Thus, we retained 171 studies, published between 1962 and 2018, that directly examined ungulate grouping behavior (see Table S1).

**FIGURE 3:**
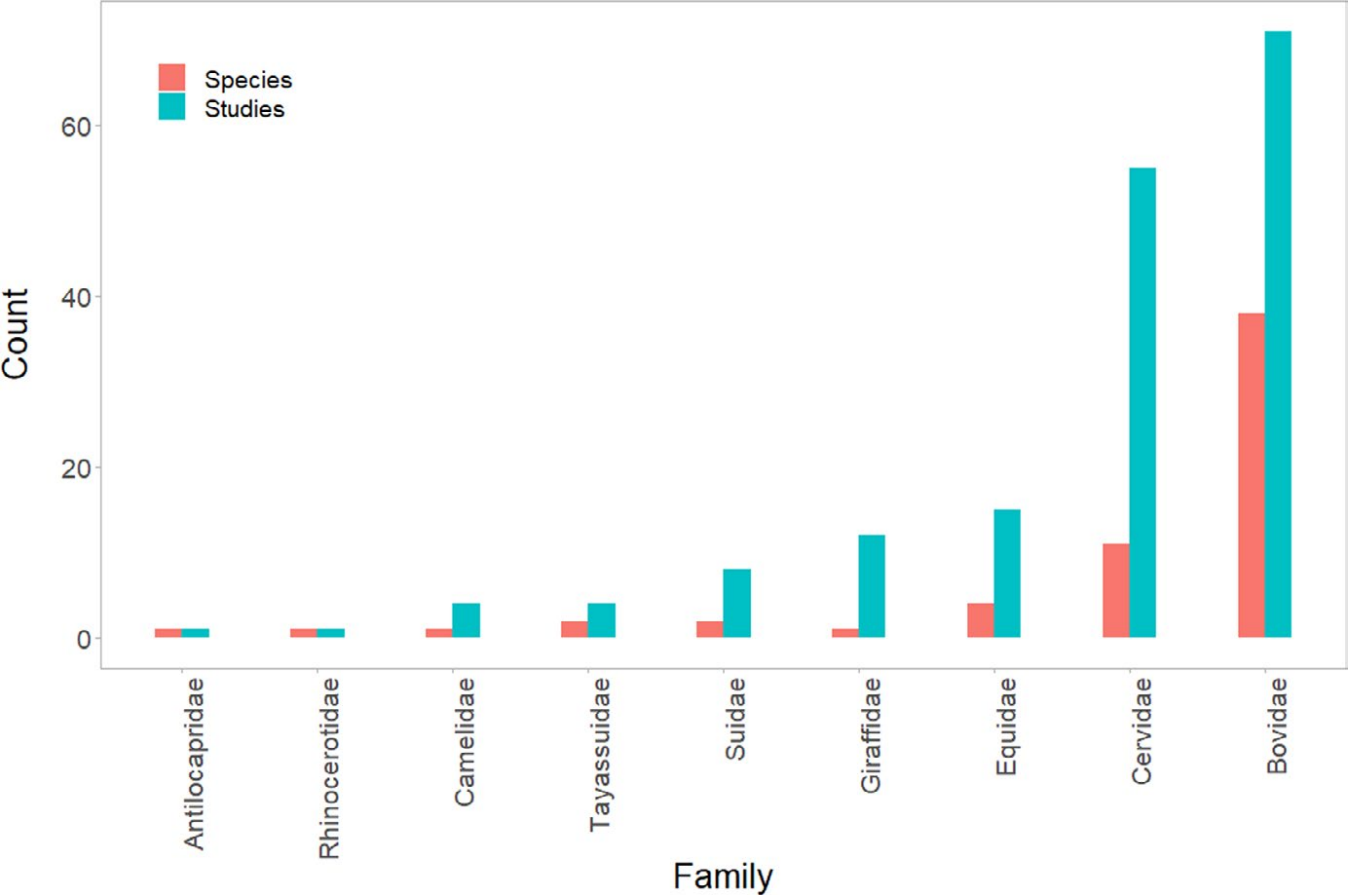
The number of species and corresponding number of studies across ungulate families featured as research subjects among 171 studies, published between 1962 and 2018, assessing ungulate group size dynamics

These 171 studies researched 61 different ungulate species among nine taxonomic families (Figure 3; Table S2). Bovids were the most common research subjects comprising 38 species across 71 (41.5%) studies. Giraffids (one species; *n* = 12 studies, 7% of all studies), Suids (two species; *n* = 8 studies, 4.7% of all studies), and Tayassuids (two species; *n* = 4 studies, 2.3% of all studies) were among the least common research subjects (Figure 3; Table S2). Our review returned no studies referencing grouping patterns in the remaining terrestrial ungulate families (i.e., Hippopotamidae and Moschidae). Approximately 92% of the studies (*n* = 158) focused their group size estimation on one species, whereas the others (*n* = 13) focused on two species or more.

When evaluated in relation to the objective categories within our framework (see Figure 2), studies referencing ungulate group sizes most often investigated social behavior (*n* = 103 studies) followed by animal‐habitat relationships (*n* = 64 studies). Studies examining predation risk (*n* = 10 studies) and disease/parasite prevalence (*n* = 4 studies) were the least common. Many studies conducted their research across more than one objective category. These included animal‐habitat relationships and social dynamics (*n* = 18 studies), animal‐habitat relationships and predation risk (*n* = 3 studies), social dynamics and predation risk (*n* = 12 studies), social dynamics and disease/parasite prevalence (*n* = 3 studies), and animal‐habitat relationships and social organization and predation risk (*n* = 3 studies).

Approximately 60% (*n* = 103 of 171) of the studies provided a formal group size definition for the ungulate species studied. The majority (91%, *n* = 93 studies) of these group definitions were derived from field observations. Only eight studies based their group definitions on telemetry data and two studies derived their group definitions from statistical reconstruction (i.e., using k‐means clustering and overlapping home ranges among individuals). Only two studies considered a group to consist of two phylogenetically close heterospecific species, whereas the rest of the studies quantified group size within a single species.

**FIGURE 4:**
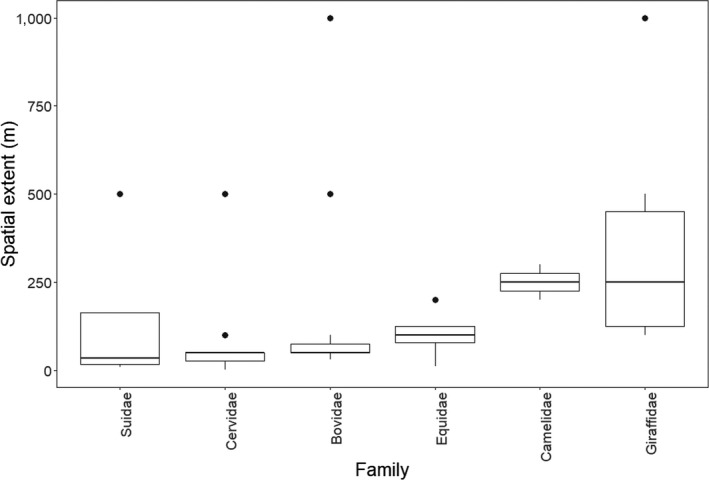
The threshold nearest neighbor distances (spatial extent) used to define group sizes of ungulate species across different families (Bovidae, *n* = 71 studies; Cervidae, *n* = 55 studies; Giraffidae, *n* = 12 studies; Camelidae, *n* = 4 studies; Suidae, *n* = 8 studies; Equidae, *n* = 15 studies) from a review of 171 studies, published between 1962 and 2018, assessing ungulate group size dynamics

These studies based their group size definitions upon the calculation or description of behavioral cohesion among group members across space and time (Table 1; Figure 4). For instance, 79.6% (*n* = 82 studies) of the group definitions emphasized a spatial extent (nearest neighbor distance) within which group members interacted (Table 1; Figure 4). The rest of the group definitions were purely subjective (*n* = 18 studies). Some group definitions, 35.9% (*n* = 37 studies) required that group members be involved in one or more similar or coordinated activities such as movement in the same direction, foraging, or under similar predation risk (inferred based on structural attributes of the environment). Only 6.8% (*n* = 7 studies) of all group definitions specified an observation time component in their group definition. This is the cutoff time in which ungulate study subjects needed to be interacting during field observation to be considered part of the same group. These times ranged from three minutes to 24 hr.

Studies expressed spatial extent as Euclidean distance in meters (94%, *n* = 77 studies, median = 50 m, range 1.4–1,000 m), or body lengths (bl) of the ungulate research subjects (*n* = 5 studies, median = 6 bl, range 1–10 bl). Of the studies that specified spatial extent in meters, the majority (41.6%, *n* = 32 studies) used 50 m as a cutoff to delineate group sizes. Studies of species in the family Bovidae had an average spatial extent of 120.67 m (median = 50 m, range 30–1,000 m), whereas Camelids had an average of 212.5 m (median = 300 m, range 50–300 m), and Giraffids had an average of 366.67 m (median = 250 m, range 100–1,000 m, Figure 4).

**FIGURE 5:**
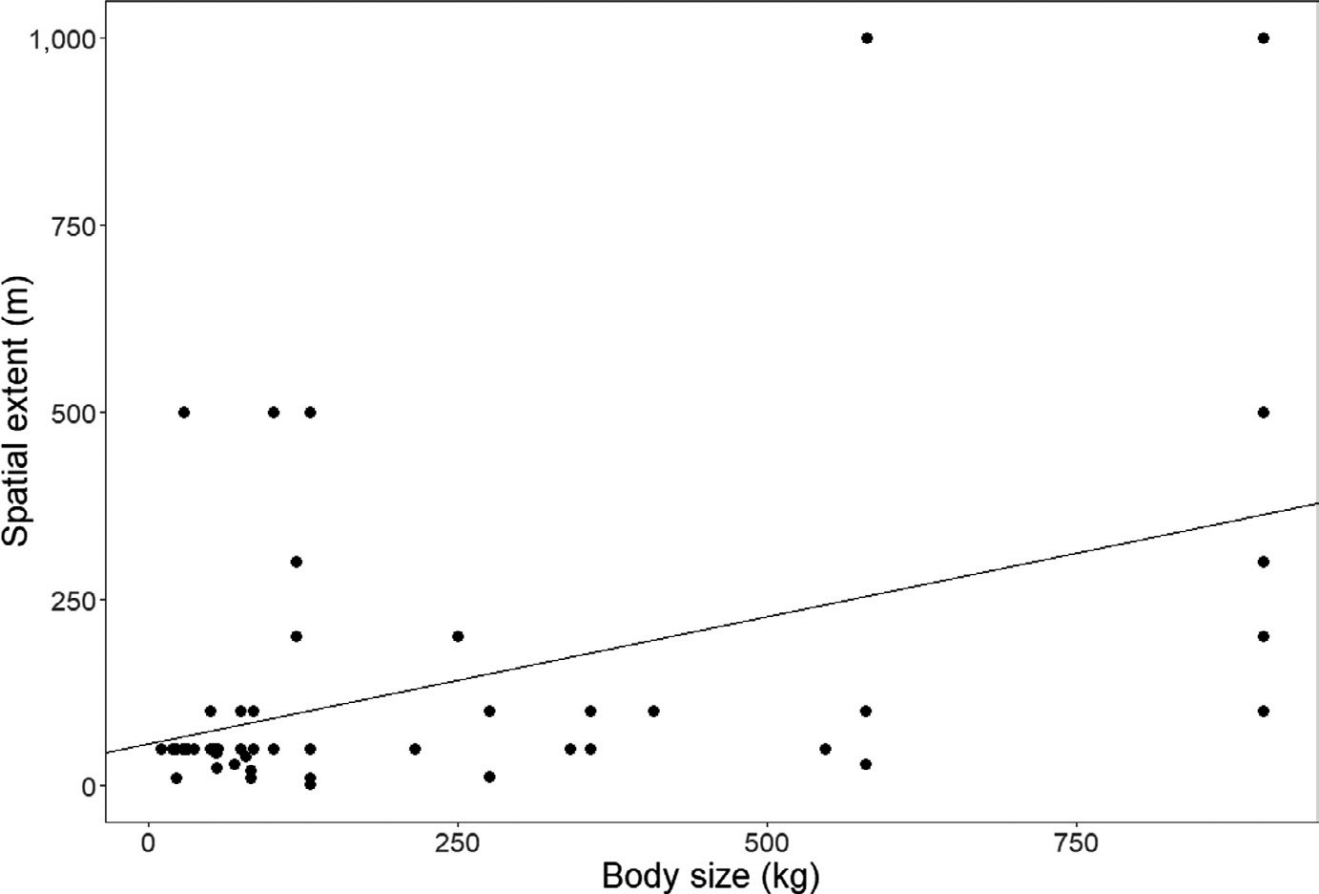
The relationship between ungulate body size and spatial extent used to define ungulate group sizes from a review of 171 studies, published between 1962 and 2018, assessing ungulate group size dynamics

We detected a weak positive correlation between body size of the research species and the spatial extent used to define their group sizes (|*r*| = .4, *p* < .003, Spearman's rank correlation, Figure 5).

## 4 DISCUSSION

Our review highlights the striking variation in group size definitions for 61 ungulate species among 171 peer‐reviewed studies published over the past six decades. These studies investigated predation risk, animal‐habitat relationships, social behavior, and disease/parasite prevalence in ungulates with group size as a fundamental requirement for drawing inference among each of these objective categories. Depending on their respective goals, studies defined ungulate group sizes from field observations (e.g., Alados, 1985; Mooring et al., 2005), telemetry data (e.g., Deacon & Bercovitch, 2018; Wal, Laforge, & McLoughlin, 2014), or statistical reconstruction (e.g., Hebblewhite & Pletscher, 2002; Stanley & Dunbar, 2013). A common theme among group size definitions from all studies was the description and/or calculation of the factors affecting behavioral cohesion of group members across spatiotemporal dimensions.

Studies based their group size definitions on rates of individual movements among group members, interindividual distances, properties of attraction, and synchrony of behavior (Caro, 2005; Dagg, 2014; Gerard & Richard‐Hansen, 1992; Krause & Ruxton, 2002; Miquelle, Peek, & Ballenberghe, 1992; Salazar et al., 2016). We detected considerable variation in the metrics used to define ungulate group sizes across these dimensions (i.e., spatial extent of group members and observation time; Figure 4). Furthermore, we detected a weak positive correlation between body size and spatial extent (Figure 5). Groups for large species that occupy large areas such as giraffes (*Giraffa camelopardalis*) tended to be defined by higher spatial extents than smaller species during field observations (Figure 4). This aligned with our expectation that spatial extent used to define group size positively correlates with species body size. Correspondingly, even fewer studies specified the temporal window over which they estimated group sizes. Spatial extent and observation time are important factors given that they delimit the extent over which group sizes are enumerated (Altmann, 1974; Jarman, 1974). Observation time bounds are critical because individuals under observation are active entities that switch between activities across space and time. Thus, a group defined within a given period reflects a partial record of the behavior of the animals under observation (Altmann, 1974). This is especially important in identification of subgroups within large groups, defining grouping rates, and consequently understanding fission‐fusion dynamics.

Across studies, the empirical and ecological rationale used to justify choice of spatial and temporal extents used to define ungulate group sizes was rare (e.g., Balmford, 1992; Bowyer et al., 2001; Marino, 2010). Specifically, the only rationale we found for choice of a spatial extent was constrained by technical designs of telemetry equipment (see Wal et al., 2014; Wal, Paquet, et al., 2012; Wal, Paquet, Messier, & McLoughlin, 2013; Wal, Yip, & McLoughlin, 2012) rather than the ecology of species under study. These studies programmed proximity‐logging collars to activate and collect data on elk (*Cervus elaphus*) interaction rates when the collared elk individuals came within 1.4 m of each other (Wal et al., 2013, 2014; Wal, Paquet, et al., 2012; Wal, Yip, et al., 2012). Unlike distance, time bound employed for group definitions derived from telemetry studies was not constrained by technical design of telemetry equipment (Wal et al., 2013, 2014; Wal, Paquet, et al., 2012; Wal, Yip, et al., 2012). Overall, uncertainty to the ecological or empirical basis for the choice of spatial and temporal extents for defining ungulate group sizes remains.

The lack of empirical and ecological rationale for choice of spatial extent and observation time for ungulate group size estimation challenges cross‐system comparisons and problematizes the development of theory. Spatial extent is critical when there is clear separation among groups with little to no observable movement of individuals among the groups (Cross, Lloyd‐Smith, & Getz, 2005). Within this context, distance‐based group definitions may be problematic for characterizing herds for highly active species. For instance, highly mobile species may perceive groups at larger spatial scales than less mobile and sedentary species (Cross et al., 2005). Additionally, large spatial extent may be limited by detectability of animal groups across large spatial scales and may only be suited to air surveys for species that occur in very large congregations. We found only one study that restricted its group definition to the number of individuals within sight of the observer(s) (see Bercovitch & Berry, 2014).

Additionally, the subjectivity inherent among ungulate group size definitions may make studies susceptible to observer bias. While subjective criteria such as “involvement in same general activity,” “awareness among group members,” and “movement in the same general direction” are not implausible, they are hard to practically contextualize among several observers, species, survey periods, or across study sites. We suggest that definitions of ungulate group sizes be rooted in empirical foundational structures with clearly articulated descriptions that can be readily replicated across studies. This is particularly important for the comparison of animal ecology across species and study sites. For example, the species included in our analysis inhabit different habitats in different locations (Figure 1) and adopt a variety of social behaviors. For instance, the beira (*Dorcatragus megalotis*) is a rare antelope that mostly lives in very small mixed‐sex family groups in arid mountainous ecosystems in East Africa (Giotto, Laurent, Mohamed, Prevot, & Gerard, 2008). In contrast, the caribou/reindeer (*Rangifer tarandus*) is a highly gregarious species with a circumpolar distribution extending across northern North America and Europe (Gunn, 2016). While the group size definitions of these two species might logically vary, there is little utility in having intraspecies variation as well. Furthermore, interspecies variation in life history traits and range dynamics complicate efforts to make species‐level inferences for ecological and conservation purposes.

## 5 CONCLUSIONS

We have highlighted considerable variability in the ways in which researchers examined ungulate group sizes across species and study systems. A critical consequence of this variability is that it may hinder the comparability and replicability of studies. To facilitate comparison, replicability of experimental designs, and evaluation of the underlying theories, researchers will need to move toward coherence in the ways they define ungulate groups. Thus, we recommend that researchers clearly describe their empirical structures for determining spatial and temporal extents used to define ungulate groups. Furthermore, studies should seek to align spatiotemporal extents used to define group sizes among individual species to facilitate cross‐system comparisons of ungulate group size dynamics. Future analyses may conduct empirical assessments of the extent to which variation in group size definitions affects resultant inference across species and study contexts. Such studies would provide further insights into the consequences of subjectively defining ungulate groups. These efforts would further illuminate the need for coherence in design and definition of ungulate groups. We concede that a uniform group definition may not be practical across all ungulate species and study contexts (sensu Krause & Ruxton, 2002), but clear rationale and foundational structures for defining groups are a critical need. While our review focused on ungulates, we believe that our recommendations are equally applicable to group‐living mammals from other taxonomic groups.

## CONFLICT OF INTEREST

The authors report no conflict of interest.

## AUTHOR CONTRIBUTIONS

**Herbert Kasozi:** Conceptualization (lead); Data curation (lead); Formal analysis (lead); Funding acquisition (equal); Investigation (lead); Methodology (lead); Project administration (equal); Validation (equal); Writing‐original draft (lead); Writing‐review & editing (lead). **Robert A. Montgomery:** Conceptualization (supporting); Data curation (supporting); Formal analysis (supporting); Funding acquisition (equal); Investigation (supporting); Methodology (supporting); Project administration (equal); Validation (equal); Writing‐original draft (supporting); Writing‐review & editing (supporting).

## Supporting information

Supplementary Material

Supplementary Material

## Data Availability

Supplementary data are provided in the supporting information.
