# Supplementary material for: Variability in the estimation of ungulate group sizes complicates ecological inference
Source: Ecol Evol. 2020 Jun 10;10(14):6881–9. doi: 10.1002/ece3.6463 (PMC7391342; doi:10.1002/ece3.6463)
Supplement: Supplementary file 2 — Supplementary Material [file ECE3-10-6881-s002.docx]

**VARIABILITY IN THE ESTIMATION OF UNGULATE GROUP SIZES COMPLICATES ECOLOGICAL INFERENCE**

HERBERT KASOZI^1^*, [kasozihe@msu.edu](mailto:kasozihe@msu.edu)

ROBERT A. MONTGOMERY^1^, [montg164@msu.edu](mailto:montg164@msu.edu)

^1^Research on the Ecology of Carnivores and their Prey (RECaP) Laboratory, Department of Fisheries and Wildlife, Michigan State University, 480 Wilson Road, 13 Natural Resources Building, East Lansing, MI  48824, USA.

**Supplementary Table S2.** Ungulate families and species identified as research subjects from 171 studies published between 1962 and 2018 describing ungulate group size dynamics. The nearest neighbor threshold distances used to define groups (Euclidean distance or body length - BL) and field data collection technique (DO – Direct Observation, T – Telemetry, CT – Camera Trapping) for each species.

| Family/  Species | Common Name | Nearest Neighbor  Threshold (m) | Group Data Collection | No. of studies | References | | |
| --- | --- | --- | --- | --- | --- | --- | --- |
| *Antilocapridae* |  |  |  |  |  | | |
| *Antilocapra americana* | Pronghorn | NA | DO | 1 | Kitchen (1974) | | |
| *Bovidae* |  |  |  |  |  | | |
| *Aepyceros melampus* | Impala | 50 | DO | 6 | Dasmann and Mossman (1962); Fritz and Garine-Wichatitsky (1996); Shorrocks and Cokayne (2005); Averbeck et al. (2009); Pays et al. (2012); Favreau et al. (2013) | | |
| *Antidorcas marsupialis* | Springbok | 50 | DO | 1 | Siegfried (1980) | | |
| *Antilope cervicapra* | Blackbuck | 50 | DO | 2 | Isvaran (2005), (2007) | | |
| *Bison bison* | American Bison | 30, 100 | T | 6 | Komers et al. (1993); Fortin et al. (2003), (2009); Green (1992); Mooring et al. (2005); Bowyer et al. (2007) | | |
| *Bison bonasus* | European bison | NA | DO | 1 | Krasinska and Krasinski (1995) | | |
| *Bos mutus* | Yak | 50 | DO | 1 | Berger et al. (2014) | | |
| *Capra hircus* | Feral goats | NA | DO | 2 | Stanley and Dunbar (2013);  Leidy et al. (2015) | | |
| *Capra ibex* | Ibex | 50, 100 | DO, T | 5 | Gross et al. (1995);  Villaret and Bon (1998);  Ruckstuhl and Neuhaus (2001);  Grignolio et al. (2007); Scillitani et al. (2013) | | |
| *Capra falconeri* | Markhor | NA | DO | 1 | Ahmad et al. (2018) | | |
| *Capra pyrenica* | Spanish Ibex | 50, 100 | DO | 1 | Aldos 1985 | | |
| *Connochaetus taurinus* | Wildbeest | 6 BL | DO | 3 | Talbot and Talbot (1963);  Scheel (1993); Thaker et al. (2010) | | |
| *Damaliscus lunatus* | Topi | NA | DO | 1 | Bro-Jorgensen (2003) | | |
| *Dorcatragus megalotis* | Beira | 50 | DO | 2 | Giotto et al. (2008);  Giotto and Gerard (2010) | | |
| *Gazella dorcas neglecta* | Dorcas gazelle | NA | DO | 1 | Abaigar et al. (2016) | | |
| *Gazella* | Mountain gazelle | NA | DO | 1 | Manor and Saltz (2003) | | |
| *Gazella granti* | Grant's Gazelle | 50 | DO | 1 | Fitzgibbon (1990) | | |
| *Gazella subgutturosa* | Goitered Gazelle | 50 | DO | 1 | Blank et al. (2012) | | |
| *Gazella thomsoni* | Thomson's Gazelle | 50 | DO | 2 | Borner et al. (1987);  Fitzgibbon (1990) | |  |
| *Hippotragus niger variani* | Sable Antelope | NA | DO | 1 | Estes and Estes (1974) | |  |
| *Kobus ellipsiprymnus defassa* | Waterbuck | NA | DO | 1 | Pays et al. (2007) | |  |
| *Kobus kob thomasi* | Uganda Kob | 40 | DO | 1 | Balmford (1990) | |  |
| *Oryx gazelle* | African oryx | NA | DO | 1 | Ruckstuhl and Neuhaus (2009) | |  |
| *Ourebia ourebi* | Oribi | NA | DO | 1 | Brashares and Arcese (2002) | |  |
| *Ovibos moschatus* | Muskoxen | 50 | DO | 1 | Ihl and Boyer (2011) | |  |
| *Ovis ammon hodgsoni* | Tibetan Argali | NA | DO | 1 | Singh et al. (2010) | |  |
| *Ovis aries* | Soay sheep | NA | DO | 3 | Coulson et al. (1999); Clutton-Brock and Coulson (2002); Pereze-Barberia and Walker (2018) | |  |
| *Ovis canadensis* | Big horn sheep | 50, 100 | DO, T | 4 | Bleich et al. (1997); Ruckstuhl (1999); Meldrum and Ruckstuhl (2009); Schroeder et al. (2010) | |  |
| *Ovis dalli* | Dall's sheep | NA | DO | 1 | Frid 1997 | |  |
| *Ovis orientalis* | Cyprus mouflon | NA | DO | 1 | Maisels 1993 | |  |
| *Procapra picticaudata* | Tibetan gazelle | 50 | DO | 1 | Li et al. 2010 | |  |
| *Procapra przewalskii* | Przewalski's gazelle | 50 | DO | 4 | Li et al. (2009), (2010), (2012);  Shi et al. (2011) | |  |
| *Rupicapra pyrenaica* | Pyrenean chamois | 50 | DO | 3 | Richard-Hansen and Campan (1992);  Gerard and Richard-Hansen (1992);  Dalmau et al. (2010) | |  |
| *Saiga tatarica* | Saiga antelope | 500 | DO, T | 2 | Milner-Gulland (2001);  Buuveibaatar et al. (2013) | |  |
| *Syncerus caffer* | African buffalo | 1000 | T | 1 | Cross et al. (2005) | |  |
| *Tragelaphus buxtoni* | Mountain nyala | 50 | DO | 1 | Tadesse and Kotler (2014) | |  |
| *Tragelaphus eurycelus* | Bongo | NA | DO | 1 | Turkalo and Klaus-Hugi (1999) | |  |
| *Tragelaphus spekei* | Sitatunga | NA | DO | 1 | Games (1983) | |  |
| *Tragelaphus strepsiceros* | Greater Kudu | NA | DO | 2 | Owen-Smith (1993); Perrin (1999) | |  |
| *Camelidae* |  |  |  |  |  |  |  |
| *Lama guanicoe* | Guanaco | 200, 300 | DO, T | 4 | Marino and Baldi (2008), (2014); Marino (2010);  Taraborelli et al. (2012) |  |  |
| *Cervidae* |  |  |  |  |  |  |  |
| *Alces alces* | Moose | 50, 100 | DO, T | 3 | Miquelle et al. (1992);  Molvar and Bowyer (1994);  Mansson et al. (2017) |  |  |
| *Capreolus capreolus* | Roe deer | 10, 50 | DO | 5 | Cibien et al. (1989); Wei-Dong et al. (2005); Pays et al. (2007); Barja and Rosellini (2008); Ferretti et al. (2011); |  |  |
| *Cervus canadensis* | Elk | 1.4, 500 | DO, T, CT | 6 | Wal et al. (2012 a, b), (2013), (2014); Brennan et al. (2014), (2015) |  |  |
| *Cervus elaphus* | Elk | 1.4, 10, 50 | DO, T | 13 | Bender and Haufler (1996); Weckerly (1999); Weckerly (2001 a, b); Clutton-Brock and Coulson (2002); Hebblewhite and Pletscher (2002); Creel and Winnie, Jr (2005); Wlodimierz et al. (2006); Barja and Rosellini (2008); Hebblewhite and Merrill (2011); Bonenfant et al. (2012); Wal et al. (2012 a); Peterson and Weckerly (2017) |  |  |
| *Cervus eldi thamin* | Thamin | NA | DO, T | 1 | Aung et al. (2011) |  |  |
| *Dama* | Fallow deer | 50 | DO | 3 | Apollonio et al. (1998); Focardi and Pechhioli (2005); Ferretti et al. (2011) |  |  |
| *Hippocamelus bisulcus* | Huemul | 30 | DO | 2 | Frid (1994), (1999) |  |  |
| *Hyelaphus porcinus* | Hog deer | NA | DO | 1 | Dhungel and O'gara (1991) |  |  |
| *Odocoileus hemionus* | Deer | 45, 50, 10 BL | DO, T | 6 | Anthony and Smith (1977); Bender (2000); Bowyer et al. (2001 a, b);  Lingle (2002); Salazar et al. (2016) |  |  |
| *Odocoileus virginianus* | White-tailed deer | 25, 45, 50 | DO, CT, T | 7 | Anthony and Smith (1977); Hirth (1977); Gavin et al. (1984); Lagory (1987); Lingle (2002); Cherry et al. (2015); Koen et al. (2017) |  |  |
| *Rangifer tarandus* | Reindeer | NA | DO, T | 7 | Bergerud (1971); Barten et al. (2001); Solberg et al. (2001); Reimers et al. (2009); L'Italien et al. (2012); Body et al. (2015 a, b) |  |  |
| *Equidae* |  |  |  |  |  |  |  |
| *Equus ferus* | Horse | 1 BL, 2 BL, 200 | DO | 5 | Duncan (1979); Rubenstein (1994); Kaczensky et al. (2008); Cameron et al. (2009); Stanley et al. (2018) |  |  |
| *Equus grevyi* | Grevy's zebra | 100 | DO | 2 | Rubenstein (2010) |  |  |
| *Equus hemionus* | Onager | NA | DO | 2 | Rubenstein (1994); Kaczensky et al. (2008) |  |  |
| *Equus quagga burchelli* | Burchell's zebra | 12, 6 BL, 100 | DO | 6 | Rubenstein (1994); (2010); Georgiadis et al. (2003); Thaker et al. (2010); Schmitt et al. (2014), (2016) |  |  |
| *Giraffidae* |  |  |  |  |  |  |  |
| *Giraffa camelopardalis* | Giraffe | 100, 200, 300, 500, 1000 | DO, T | 12 | Foster and Dagg (1972); van der Jeugd and Prins (2000); Shorrocks and Croft (2009); VanderWaal et al. (2014); Schmitt et al. (2016); Deacon and Bercovitch (2018); Bercovitch and Berry (2009), (2013), (2014), (2018); Muller et al. (2018a, b) |  |  |
| *Rhinocerotidae* |  |  |  |  |  |  |  |
| *Ceratotherium simum* | White Rhino | NA | DO | 1 | Shrader et al. (2013) |  |  |
| *Suidae* |  |  |  |  |  |  |  |
| *Phacochoerus africanus* | Warthog | 10, 20, 6 BL | DO | 6 | Scheel (1993); White et al. (2010); White (2010); White and Cameron (2011a, b, c) |  |  |
| *Sus scrofa* | Wild boar | 50, 500 | DO, T | 2 | Iacolina et al. (2009); Ferretti et al. (2011) |  |  |
| *Tayassuidae* |  |  |  |  |  |  |  |
| *Tayassu pecari* | White-Lipped Peccary | NA | T | 2 | Fragoso (1998); Reyna-Hurtado et al. (2016) |  |  |
| *Tayassu tajacu* | Collared Peccary | NA | DO | 2 | Byers and Bekoff (1981); Gabor and Hellgren (2000) |  |  |
